# Reply to Eker et al. Comment on “Kilian et al. Comparing Characteristics and Treatment of Brain Vascular Malformations in Children and Adults with HHT. *J. Clin. Med.* 2023, *12*, 2704”

**DOI:** 10.3390/jcm12237462

**Published:** 2023-12-01

**Authors:** Alexandra Kilian, Giuseppe A. Latino, Andrew J. White, Felix Ratjen, Jamie McDonald, Kevin J. Whitehead, James R. Gossage, Timo Krings, Michael T. Lawton, Helen Kim, Marie E. Faughnan, The Brain Vascular Malformation Consortium HHT Investigator Group

**Affiliations:** 1 Department of Paediatrics, The Hospital for Sick Children, Toronto, ON M5G 1X8, Canada; 2 Toronto HHT Centre, St. Michael’s Hospital, Li Ka Shing Knowledge Institute, Toronto, ON M5B 1W8, Canada; 3 Department of Pediatrics, North York General Hospital, University of Toronto, Toronto, ON M2K 1E1, Canada; 4 Department of Pediatrics, St Louis University, St. Louis, MO 63103, USA; 5 Division of Respiratory Medicine and Translational Medicine, The Hospital for Sick Children, Toronto, ON M5G 1X8, Canada; 6 Department of Pathology, University of Utah, Salt Lake City, UT 84132, USA; 7 Department of Medicine, Division of Cardiovascular Medicine, University of Utah, Salt Lake City, UT 84132, USA; 8 Department of Pediatrics, Division of Pediatric Cardiology, University of Utah, Salt Lake City, UT 84132, USA; 9 Department of Medicine, Augusta University, Augusta, GA 30912, USA; jgossage@augusta.edu; 10 Division of Neuroradiology, Toronto Western Hospital, University Health Network, Toronto, ON M5T 2S8, Canada; timo.krings@uhn.ca; 11 Department of Neurosurgery, Barrow Neurological Institute, Phoenix, AZ 85013, USA; 12 Center for Cerebrovascular Research, Department of Anesthesia and Perioperative Care, University of California San Francisco, San Francisco, CA 94110, USA; 13 Institute for Human Genetics, University of California San Francisco, San Francisco, CA 94143, USA; 14 Department of Epidemiology and Biostatistics, University of California San Francisco, San Francisco, CA 94158, USA; 15 Division of Respirology, Department of Medicine, University of Toronto, Toronto, ON M5S 3H2, Canada

We are grateful to Eker et al. for their thoughtful analysis and response to our publication titled Comparing Characteristics and Treatment of Brain Vascular Malformations in Children and Adults with HHT [1]. We welcome the opportunity to further discuss this important topic.

Eker et al. identify several important methodological limitations, including the fact that our cohort is “highly selected” and that our analysis did not include brain VM subtypes. We agree that our results and conclusions must be interpreted considering their limitations. We explicitly acknowledged these same limitations in the discussion and limitation section of our manuscript and would direct readers to those sections for additional details.

As stated in our limitations section, the targeted recruitment of patients with brain VMs in the BVMC HHT project may predispose to the recruitment of symptomatic patients or patients with more severe disease, which may impact the treatment trends seen in our data [2]. Basing themselves on this, Eker et al. suggest that the prevalence of intracranial hemorrhage (ICH) that we report is “not comparable to previously reported data on larger based HHT-populations.” In response, we would direct readers to our discussion section that explores the literature to date that has attempted to quantify the risk, incidence and prevalence of ICH and rupture in HHT-related brain VMs. Overall, this has proven to be a challenging question. As we did in our discussion, we would also like to draw the readers’ attention to the systematic review by Brinjikji et al., which concluded that approximately 20% of HHT patients with brain VMs will suffer an ICH [3]. Thus, the prevalence of ICH reported in our data (23.7% among pediatric patients with HHT-related brain VMs and 9.9% among adult patients with HHT-related brain VMs) aligns with previously reported data.

Eker et al. suggest that it would have been more appropriate to present “the proportions of ruptured and unruptured VMs among the treated patients” rather than present the proportions of treated brain VMs within the ruptured and unruptured groups as we had done in our manuscript. We are appreciative of the group’s thoughtful analysis and agree that both presentations of the data provide valuable information on understanding the prevalence of rupture and treatment trends. The high proportion of unruptured VMs treated in the pediatric population is an important treatment trend to be aware of, as revealed by our data and highlighted by Eker et al.

We are keen for future research endeavors to explore current clinical justifications for treating unruptured brain VMs, as thoughtfully proposed by Eker et al. Valuable future research directions also include a detailed analysis of post-treatment hemorrhages and complications in treated patients, as suggested by Eker et al.

Ultimately, the issue of screening for brain VMs in children with HHT is a complex, nuanced clinical question that requires thoughtful consideration by both individual clinicians and the HHT patient community, as well as ongoing data and research. Given the nuance and complexity associated with this question and the importance of data in facilitating evidence-based clinical decision making for patients with HHT, we felt it was important to make this large series on the topic accessible to clinicians and the HHT patient community while acknowledging its limitations. We look forward to future research that helps address the many still unanswered questions in the field, some of which were highlighted by Eker et al.
